# Pigmented Villonodular Synovitis of the Knee Joint: A Case Report

**DOI:** 10.7759/cureus.816

**Published:** 2016-10-04

**Authors:** Chirag Kapoor, Maulik Jhaveri, Rishit Soni, Malkesh Shah, Parth Rathi, Paresh Golwala

**Affiliations:** 1 Orthopaedics, Sumandeep Vidyapeeth, Vadodara, Gujarat

**Keywords:** villonodular, synovitis, synovectomy, mri

## Abstract

Pigmented villonodular synovitis (PVNS) is a rare, benign, but potentially locally aggressive and recurrent condition characterized by synovial proliferation and hemosiderin deposition inside the joints, tendon sheaths, and bursae. It usually affects the large joints such as hip, knee, and ankle. We report a case of PVNS of the knee joint in a young female which was treated by subtotal synovectomy alone without the use of adjuvants. At the 14-month follow-up, the patient was pain free and had no signs of disease recurrence.

## Introduction

Pigmented villonodular synovitis (PVNS), coined by Jaffe, et al. [1] in 1941 is a rare, benign, but potentially locally aggressive and recurrent condition. It is characterized by synovial proliferation and hemosiderin deposition inside the joints, tendon sheaths, and bursae. It usually affects the large joints, i.e. hip, knee, and ankle, but few cases of PVNS involving small joints have been reported [1]. The most commonly involved joint has been the knee, followed by the hip and the ankle [2]. There are two types of PVNS: localized and diffuse. The diffuse type is reportedly three times more common than the localized type [3].

The etiology of PVNS is not certain but some researchers have debated whether it is inflammatory or neoplastic in origin while others have suggested trauma-induced hemorrhage as an etiology.

## Case presentation

**Figure 1:**
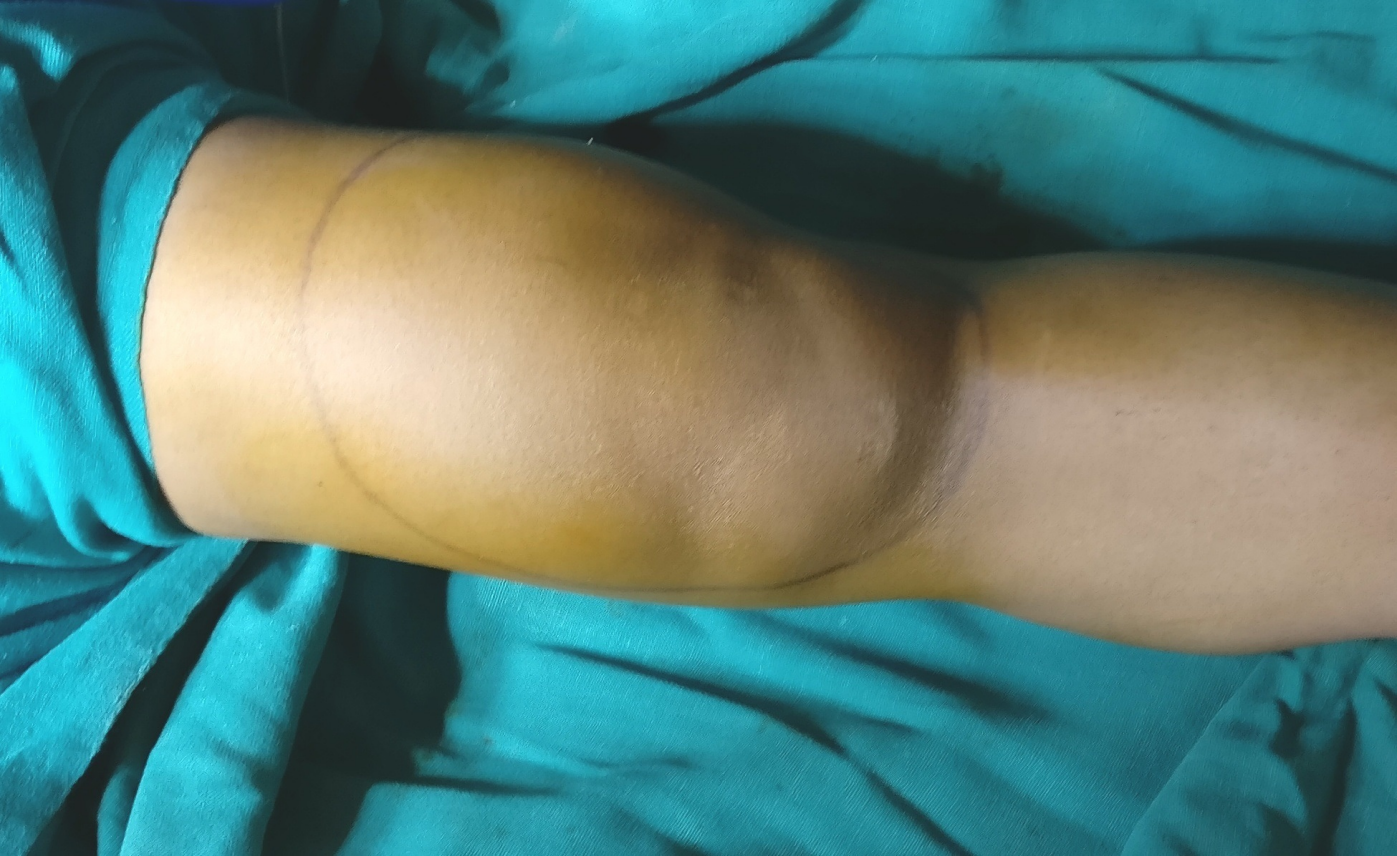
Clinical image showing the swelling around the knee joint

A 28-year-old female presented to us with a six-month history of pain and swelling in the left knee joint. The swelling gradually increased over a period of time and was associated with difficulty in walking and standing. The swelling was diffuse and nodular in consistency, measuring 17 cm x 11 cm in size (Figure 1). The overlying skin was normal with no signs of inflammation. There was no instability of the knee joint on physical examination. No other joint was involved.

**Figure 2:**
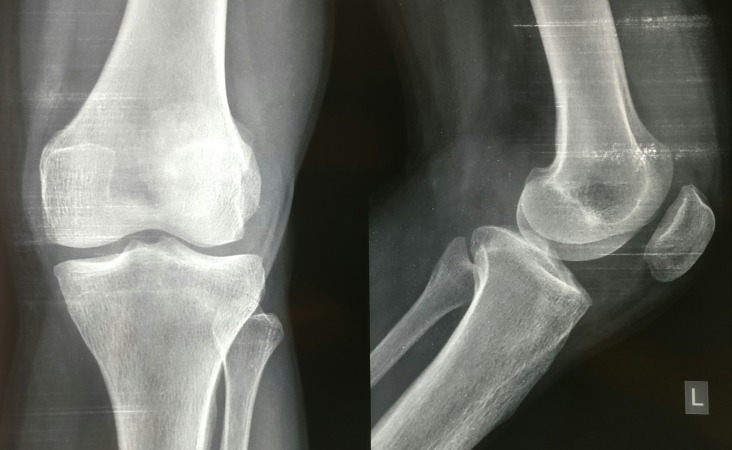
Pre-op radiograph of knee joint showing no abnormality

**Figure 3:**
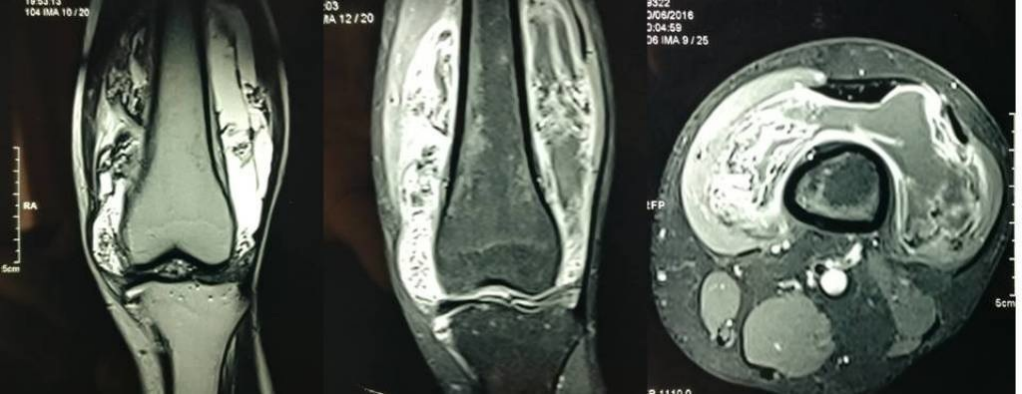
MRI of the knee joint - coronal and axial views Mulitlobulated lesions seen medially and laterally around the knee joint suggestive of PVNS.

Radiographs of the left knee were obtained which showed no bony abnormality (Figure 2). A magnetic resonance imaging (MRI) scan revealed a large joint effusion seen predominantly in the suprapatellar recess as well as in the lateral and medial femoral recesses appearing hyperintense on T2-weighted images. Diffuse synovial thickening was seen which appeared hypointense on T1 as well as hypo on T2-weighted and susceptibility images, and the synovium appeared hypertrophied. A multilobulated lesion was seen in continuity with the synovium in the anteromedial, anterolateral, and patella-femoral joint space. Few internal septae were seen. On postcontrast scan, thick enhancement was seen along the synovium (Figure 3). All these features were consistent with the diagnosis of pigmented villonodular synovitis.

**Figure 4:**
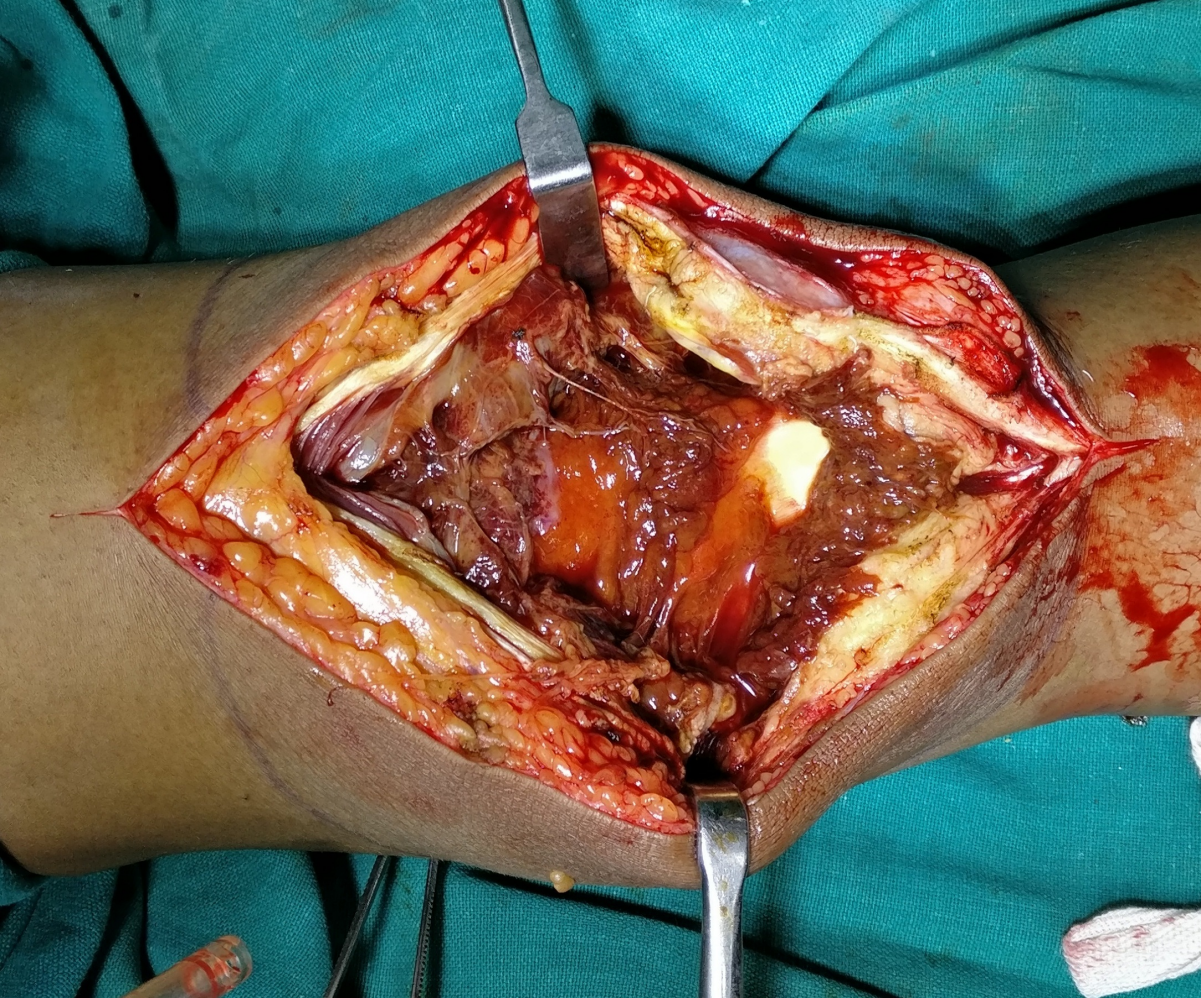
Intra-operative image Image shows multinodular brownish synovium around the knee joint.

After obtaining written informed consent from the patient, she was submitted to surgery. Sub-total synovectomy was done using a medial parapatellar approach to the knee joint. The synovium was excised in toto as a pouch. Intraoperatively, a brownish nodular synovium was found which again was suggestive of PVNS (Figure 4).

**Figure 5:**
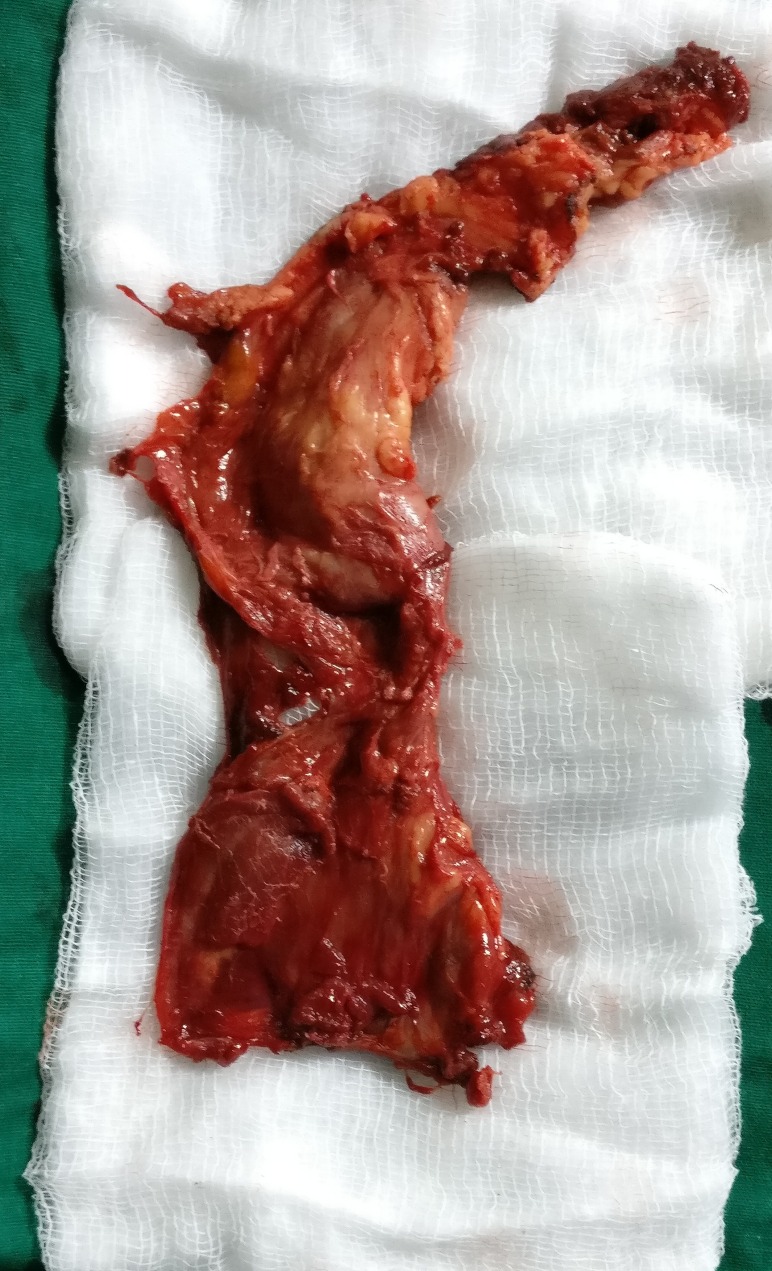
The excised tissue after synovectomy

**Figure 6:**
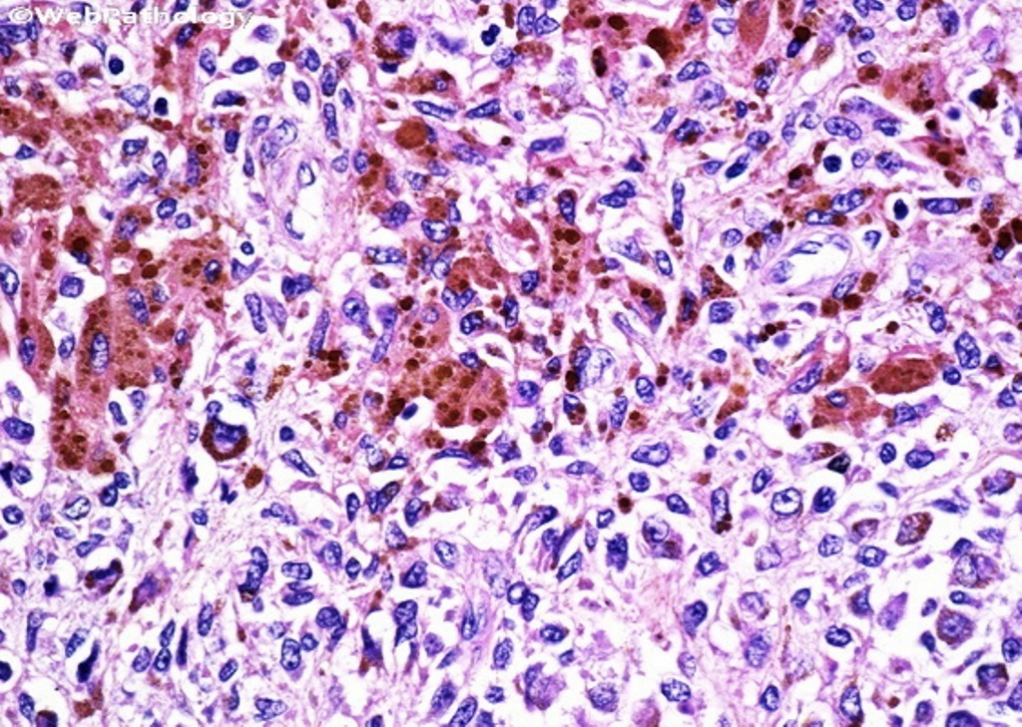
Histopathological slide showing hyperplastic synovial lining with scattered hemosiderin deposits

The excised synovium (Figure 5) was sent for histopathological examination which showed synovial tissue with hyperplastic synovial lining forming papillary proliferation with oedematous stroma and presence of granulation tissue. The blood vessels were dilated and congested, surrounded by dense inflammatory infiltrate of plasma cells, lymphocytes, and histiocytes. Scattered hemosiderin granules along with hemosiderin-laden macrophages were also seen (Figure 6). All these features confirmed the diagnosis of PVNS.

Postoperatively, the patient was started on passive knee flexion and extension exercises for 15 days and made to walk after that. Follow-up was taken at regular intervals, and at the 14-month follow-up there were no signs of recurrence both clinically and radiologically, and the patient had full knee range of movement.

## Discussion

Intra-articular PVNS is an uncommon disease. The prevalence has been estimated to be 1.8 cases per million population [4]. It commonly affects individuals in the fourth and fifth decades [5]. Most cases that occur have been monoarticular but rarely, polyarticular PVNS has been reported [6].

The mechanism of bone erosion in PVNS is still unclear. Some believe that pressure within the involved joints increases because of the synovial overgrowth while others believe that the synovium releases a substance that causes bone erosion which in turn results in joint destruction [7].

The reported rate of recurrence is varied. PVNS has been reported to have a high recurrence rate, but it rarely becomes malignant [5]. Surgical excision is the preferred management for both localized and diffuse PVNS with success being dependent on complete resection with clear margins. The best treatment for diffuse PVNS is controversial. Open surgical excision has been the primary method for treating diffuse PVNS [8]. Another method is arthroscopic synovectomy which has the advantage of smaller incisions and reduced morbidity but has reported recurrence rates as high as 46%, so some authors recommend open synovectomy [5]. It is also theoretically possible that arthroscopy results in secondary seeding because of limited surgical view and joint irrigation system which probably leads to recurrence [5].

In patients with diffuse PVNS, some authors have recommended staged anterior and posterior synovectomies. The recurrence rate associated with such treatment ranges from 14% to 56% [5]. However, total synovectomy is difficult to perform and may injure the neurovascular structures adjacent to the affected synovium. Furthermore, one report suggests that total synovectomy may increase the risk of osteoarthritis, so subtotal synovectomy is preferred [8]. Non-resectable PVNS tissue also may be controlled using adjuvant therapy, such as intra-articular instillation of radioactive isotopes, which was not necessary in our case [9]. However, adjuvant radiotherapy with agents such as yttrium-90 has been associated with side effects. We did not opt for adjuvant radiotherapy as it was possible to remove the affected synovium completely in our patient and because of the potential significant risks like radionecrosis of the soft tissue and infertility involved with radiotherapy in a young female [10]. Kotwal, et al. [9] reported no recurrence of PVNS after surgery followed by postoperative radiotherapy and six percent recurrence after surgery alone.

## Conclusions

PVNS is an unusual condition with a high potential for recurrence and requires excision. If it is easily resectable, adjuvant radiotherapy is not required.
